# DNA interference and beyond: structure and functions of prokaryotic Argonaute proteins

**DOI:** 10.1038/s41467-018-07449-7

**Published:** 2018-12-04

**Authors:** Lidiya Lisitskaya, Alexei A. Aravin, Andrey Kulbachinskiy

**Affiliations:** 1 Institute of Molecular Genetics, Russian Academy of Sciences, Moscow, 123182 Russia; 2 Division of Biology and Biological Engineering, California Institute of Technology, Pasadena, CA 91125 USA

## Abstract

Recognition and repression of RNA targets by Argonaute proteins guided by small RNAs is the essence of RNA interference in eukaryotes. Argonaute proteins with diverse structures are also found in many bacterial and archaeal genomes. Recent studies revealed that, similarly to their eukaryotic counterparts, prokaryotic Argonautes (pAgos) may function in cell defense against foreign genetic elements but, in contrast, preferably act on DNA targets. Many crucial details of the pAgo action, and the roles of a plethora of pAgos with non-conventional architecture remain unknown. Here, we review available structural and biochemical data on pAgos and discuss their possible functions in host defense and other genetic processes in prokaryotic cells.

## Abstract (web summary)

In this review, Aravin and colleagues examine bacterial and archaeal Argonaute proteins, discuss their diverse architectures and their possible roles in host defense, proposing additional functions for Argonaute proteins in prokaryotic cells.

## Introduction

Small noncoding RNAs are essential players in the control of gene expression and maintenance of genome stability in both prokaryotes and eukaryotes. In eukaryotes, several classes of small noncoding RNAs regulate gene expression and protect cells against exogenous and endogenous harmful genetic elements through specific recognition of complementary RNA targets, in a group of processes collectively called RNA interference (RNAi). RNAi pathways are diverse between species, and several distinct pathways can also operate within the same organism and even single cell^1^. Despite such diversity, all RNAi processes rely on a common core complex, composed of small guide RNA tightly bound to a protein from the Argonaute (Ago) family^2–7^ (Box 1). This complex (sometimes called RNA-induced silencing complex, RISC) recognizes complementary RNA targets and either directly cleaves them through endonuclease activity of Ago (slicer activity) or performs other functions—such as cleavage-independent RNA destabilization, repression of translation and transcription—by interacting with other proteins^7,8^.

Analysis of prokaryotic genomes revealed broad distribution of Ago proteins in both archaea (~30% of all sequenced genomes) and bacteria (~10% of genomes)^9–11^. Remarkably, pAgos are much more diverse than eAgos, and the latter form only a small branch on the pAgo tree suggesting their origin from pAgos^9,10^. Structural and biochemical studies of pAgos, in particular, from thermophilic prokaryotes, revealed a detailed pathway of guide binding, target recognition and slicer activity that provided crucial insight into the molecular mechanisms of RNAi in eukaryotes. However, until recently no information about the functions of these proteins in their prokaryotic hosts was available and their natural nucleic acid partners in the cell were unknown. Here, we review available data on the complexes of various pAgos with nucleic acids, and describe known biochemical activities of pAgos. We further discuss their emerging role in the genome defense against foreign genetic elements and hypothesize that they may perform additional functions in the regulation of genetic processes (e.g., DNA transcription, replication and repair) in the prokaryotic cell.

### Box 1

Based on sequence and structural features, eukaryotic Ago (eAgo) proteins can be divided into two main clades, AGO and PIWI, with additional clades formed by worm-specific and trypanosomal Ago proteins^10,113–115^. Three main classes of small RNAs interacting with eAgos include microRNAs (miRNAs), small interfering RNAs (siRNAs), and PIWI-interacting RNAs (piRNAs). miRNAs and siRNAs share a partly common biogenesis pathway that relies on endonuclease Dicer that processes longer precursors to make mature small RNAs^116–118^. While siRNAs are processed by Dicer from exogenous or endogenous double-stranded RNA in the cytoplasm, miRNAs are processed from hairpin (pseudo-dsRNA) precursors that first have to be cleaved by another endonuclease, Drosha, in the nucleus^117,119,120^. piRNAs, found in Metazoa, are processed from longer precursors that—in contrast to miRNA and siRNA—can be single-stranded, and their biogenesis is independent of Dicer and Drosha^76–78,121^. miRNAs and siRNAs associate with AGO-clade Ago proteins. In some species like *Drosophila* miRNAs and siRNAs are predominantly sorted into different Ago proteins^122,123^, while in others including mammals they can be loaded into the same protein. piRNAs associate with the PIWI clade Ago proteins^76,78,124–126^. The resulting effector complexes then regulate the expression of host genes, suppress transposons or combat viral infection^87,127–129^.

## Structural organization of Ago proteins

**Fig. 1:**
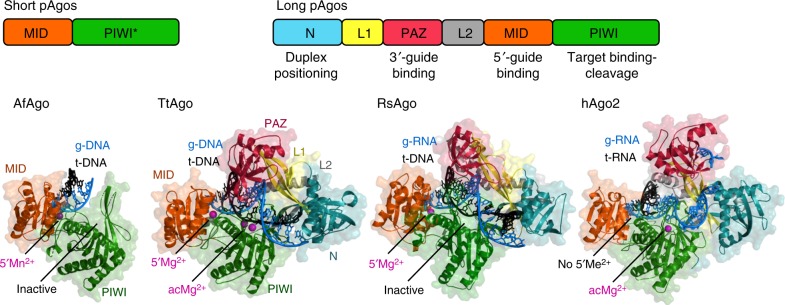
Structural organization of Ago proteins. The domain architecture of short and long pAgos is schematically illustrated at the top. Short pAgos always contain inactive PIWI domain (PIWI*). The structures of four representative Ago proteins are shown in ternary complexes with guide (“g-”) and target (“t-”) nucleic acids: short inactive AfAgo (PDB: 2W42^17^) and long active TtAgo with g-DNA and t-DNA (PDB: 4NCB^30^), long inactive RsAgo with g-RNA and t-DNA (PDB: 5AWH^35^) and active human Ago2 with g-RNA and t-RNA (PDB: 4W5O^54^). The N-domain is turquoise, L1 is yellow, PAZ is magenta, L2 is gray, MID is orange, PIWI is green. The guide strand is blue, the target strand is black. Metal ions bound in the MID-pocket (5′Me^2+^) or in the active center (acMe^2+^) are indicated

All eAgo proteins contain six structural segments, including N-terminal, L1 (Linker 1), PAZ (PIWI–Argonaute–Zwille), L2 (linker 2), MID (Middle) and PIWI (P-element Induced Wimpy Testis) domains (Fig. 1). pAgo proteins have diverse structures and can be divided into two large phylogenetic groups^9,10,12,13^. One group, denoted long pAgos, predominantly includes pAgos that contain all domains present in eukaryotic proteins, although some members of this group (e.g., AfAgo) have lost the N-PAZ domains (Fig. 1)^9,10,12^. The second group of so-called short pAgos harbors proteins that have only MID and PIWI domains. All studied eAgos and long pAgos have a bilobal structure, consisting of the N-PAZ and MID-PIWI lobes, with nucleic acids—the guide and the target—accommodated between the lobes (Fig. 1). The catalytic site is formed by the RNaseH fold of the PIWI domain; it is located in the middle of the nucleic acid binding cleft and binds two divalent metal ions for catalysis. Many pAgos, including all short pAgos, contain substitutions of essential catalytic residues suggesting that they lack endonucleolytic activity. The genes of inactive pAgos often adjoin to genes encoding putative nucleases that were proposed to play a role in biogenesis of nucleic acid guides and/or repression of their genetic targets. The genes that are next to short pAgos also always contain the APAZ (“analog of PAZ”) domain of unknown functions^9,10^.

Biochemical and structural studies of pAgo proteins from several species revealed that they can bind either DNA or RNA guides but, in contrast to eAgos, preferably recognize DNA targets (Table 1 and references therein). Several pAgos were also shown to cleave RNA targets but the functional significance of this activity remains unknown (Table 1). To date, structural models of pAgo proteins and their complexes with guide and target nucleic acids were obtained for seven proteins, including DNA-guided (AfAgo^14–17^, AaAgo^18–20^, MjAgo^21–23^, PfAgo^24–26^, and TtAgo^27–33^) and RNA-guided (RsAgo^34,35^ and MpAgo^36,37^) pAgos (see Table 1 for pAgo abbreviations). The most complete structural information was obtained for TtAgo that was crystallized with guide (DNA) and target (DNA or RNA) molecules at different steps of its functional cycle. The compendium of all published structures of pAgos and the summary of their functional properties are presented in Supplementary Fig. 1, 2, 4, Table 1 and Supplementary Table 1. For comparison, we also include eukaryotic Argonautes KpAgo^38^ (yeast *Kluyveromyces polysporus*), hAgo1^39^, hAgo2^38,40–42^, and hAgo3^43^ (human) from the AGO-clade and SIWI^44^ (silkworm *Bombyx mori*) from the PIWI-clade, all the eAgos for which three-dimensional structures have been determined to date. Below, we outline common features and structural variations observed for these proteins.

**Table 1:** Functional properties of analyzed pAgo proteins in comparison with eAgos

Host	Argonaute	Guide	5′-end; nucleotide preference	Target	Catalytic activity	Functional activity	References
*Aquifex aeolicus*	AaAgo	DNA	5′-P; Unknown	(RNA; DNA not tested)	Guide-dependent	–	^18– 20^
*Archaeoglobus fulgidus*	AfAgo (short pAgo)	DNA (RNA)	5′-P; Unknown	DNA (RNA)	Inactive	–	^14– 17^
*Marinitoga piezophila Thermotoga profunda*	MpAgo TpAgo	RNA	5′-OH; None	DNA (RNA)	Guide-dependent	–	^36, 37^
*Methanocaldococcus jannaschii*	MjAgo	DNA	5′-P; Purines	DNA	Guide-dependent; Chopping	Reduced plasmid content and transformation efficiency	^21– 23^
*Pyrococcus furiosus*	PfAgo	DNA	5′-P; None	DNA	Guide-dependent; Guide-independent	Reduced transformation efficiency	^24– 26^
*Rhodobacter sphaeroides*	RsAgo	RNA^a^	5′-P; g1U	DNA^a^	Inactive	Reduced transcription of reporter genes and plasmid content	^34, 35^
*Thermus thermophilus*	TtAgo	DNA^a^	5′-P; g1C/t1′G	DNA^a^ (RNA)	Guide-dependent; Chopping	Reduced plasmid content and transformation efficiency; changes in gene expression	^27– 33^
*Homo sapiens*	hAgo1	RNA	5′-P; g1U or g1A	RNA	Inactive	miRNA pathway	^39^
	hAgo2	RNA	5′-P; g1U/t1′A	RNA	Guide-dependent	miRNA pathway	^38, 40– 42^
*Kluyveromyces polysporus*	KpAgo	RNA	5′-P; g1U	RNA	Guide-dependent	miRNA pathway	^38^
*Bombyx mori*	SIWI	RNA	5′-P; g1U	RNA	Guide-dependent	piRNA pathway	^44^

^a^Both in vitro and in vivo

## The catalytic cycle of Ago proteins

**Fig. 2:**
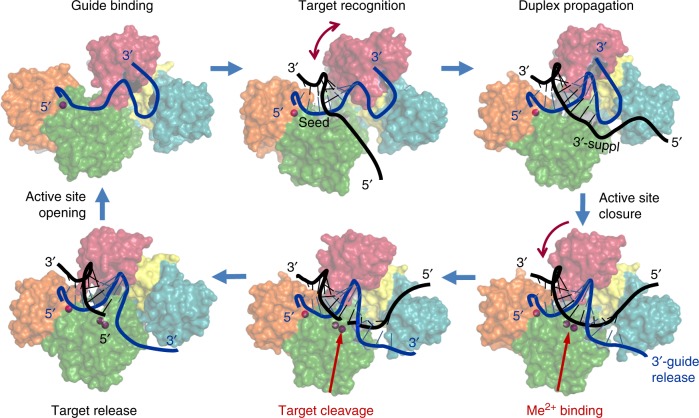
The catalytic cycle of Ago proteins. Guide-loaded Ago performs search for a complementary target through base-pairing with the seed region of the guide strand, followed by duplex propagation through the central part and the 3′-supplementary site of the guide, thus checking for possible mismatches. Conformational mobility of the PAZ domain (shown by arc-shaped arrows) likely facilitates correct base-pairing, through controlled release of the guide 3′-end and active site closure. Conformational changes in the active site allow binding of catalytic metal ions, followed by cleavage of the target strand and its stepwise release from the complex. The drawings are based on the structures of TtAgo at different steps of its functional cycle (PDBs, from the upper left corner, clockwise: 3DLH, 3F73, 4N41, 4NCB, 4NCA, 4N76, see Supplementary Fig. 2). The guide strand is blue, the target strand is black; only the target strand of DNA substrate is shown (the structure of complexes with double-stranded DNA remains unknown for any pAgo)

The main steps in the catalytic cycle of Ago proteins established in vitro include guide binding, target recognition and annealing, target cleavage and target release (Fig. 2). These steps are likely similar for catalytically active eAgos and pAgos, however, the activity cycles of various pAgo proteins may include additional functional steps, as discussed below. Catalytically inactive Agos do not cleave their targets but are similar to the active Agos in guide binding and target recognition. Molecular mechanisms of the Ago action have been covered by several recent reviews^10,12,45–47^. We therefore briefly overview the main steps of the catalytic cycle of Ago proteins with particular emphasis on pAgos.

### Guide binding and target recognition

All studied Ago proteins bind guide nucleic acid molecules (18–21 nt in analyzed structures) in a similar way, with the 5′- and 3′-ends of the guide fixed in protein pockets formed by the MID and PAZ domains, respectively (Figs. 1, 2, Supplementary Fig. 1 and Fig. 2). Analysis of eAgos suggested that the guide is subdivided into several functional segments, including the 5′ (anchor) nucleotide, the seed region (nucleotides 2–8), the site of cleavage (positions 10–11), the 3′ supplementary site (positions 12–16) and the 3′ tail, and a similar subdivision likely occurs in pAgos (Fig. 2)^12,47–50^.

Several studied pAgos have preferences for specific 5′-nucleotides in the guide molecule (Table 1), including RsAgo (prefers 5′-uridine guides, similarly to hAgo2, KpAgo and SIWI^34,35,38,40,44^), TtAgo (5′-cytosine)^31^ and MjAgo (5′-purines)^22^; other pAgos (MpAgo, PfAgo) show no 5′-end specificity^26,36^. In ternary complexes, the 5′-guide residue remains unpaired with the target and the corresponding target nucleotide (t1) can be bound and specifically recognized in a separate pocket in the PIWI domain (t1′G for TtAgo^33^, t1′A for hAgo2^41^, and RsAgo^51^).

Most pAgos bind 5′-phosphorylated guides and use a Mg^2+^ ion bound in the MID pocket for interaction with the first guide phosphate (Fig. 1, Supplementary Fig. 1; Table 1 and Supplementary Table 1). In contrast, eAgos of the AGO clade rely on a conserved lysine residue for the 5′-phosphate binding^38–40,52,53^. Unexpectedly, recent structural analysis of the silkworm SIWI protein from the PIWI clade of eAgos revealed that its MID pocket is similar to pAgos, with the Mg^2+^ ion involved in guide interactions (Supplementary Fig. 1)^44^. In contrast to other Agos, MpAgo binds unphosphorylated 5′-OH-guides and has a more hydrophobic pocket without metal ions or positively charged residues^36,37^.

In all Ago-guide complexes, several nucleotide bases from the seed region are preoriented in a helical conformation and exposed to the solution (positions 2–4 to 2–6 in various Agos^22,27,36,40^). Initial target pairing with this region induces conformational changes that expose downstream nucleotides for further target recognition (Fig. 2)^29,30,54^. The downstream part of the seed region (positions 6–8) is kinked in available structures, depending on the geometry of the nucleic acid binding cleft (Supplementary Fig. 1)^22,28,36,38–40,52^. In eukaryotic Ago2, the resulting subdivision of the seed is important for the stepwise target recognition^55^, and a similar role for guide kinking was proposed for pAgos^12^.

The 3′-proximal part of the guide, except few last nucleotides that are bound in the PAZ pocket, is disordered in all binary Ago-guide complexes suggesting that it is structurally flexible (Figs. 1, 2). This includes the 3′ supplementary site that plays an important role in the recognition of mRNA targets by eAgos^48–50^ and of DNA targets by analyzed pAgos (e.g., MpAgo^37^ and RsAgo^51^). Such flexibility may likely facilitate helix formation during target annealing.

The 3′-end of the guide is bound in the PAZ pocket in binary complexes but is extruded upon target annealing (Fig. 2, Supplementary Fig. 1 and 2)^21,29,30,37,56^. For TtAgo, the 3′-guide release was observed after formation of a 12 bp g-DNA/t-RNA duplex or a 16 bp g-DNA/t-DNA duplex (Supplementary Fig. 2), suggesting that these processes are tightly coordinated and depend on the structure of the target strand^29,30^. Indeed, the guide-PAZ interactions are important for specific target recognition^21,56,57^ and may also prevent guide degradation by cellular nucleases^57^. The PAZ pocket may exhibit certain preferences toward 3′-guide nucleotides in some pAgos (e.g., pyrimidine bases in MjAgo)^22^ but the functional importance of this remains to be investigated. The conformational mobility of the PAZ domain (indicated with arrows in Fig. 2, Supplementary Fig. 1) may also contribute to the ability of various Agos to interact with populations of short RNAs or DNAs with different length distributions. At the same time, some pAgos have an incomplete PAZ domain (RsAgo^35,51^, MpAgo^36^) or completely lack it (short pAgos, such as AfAgo, Fig. 1). It remains to be established whether additional proteins may be involved in 3′-guide interactions in such pAgos.

### Catalysis and target release

**Fig. 3:**
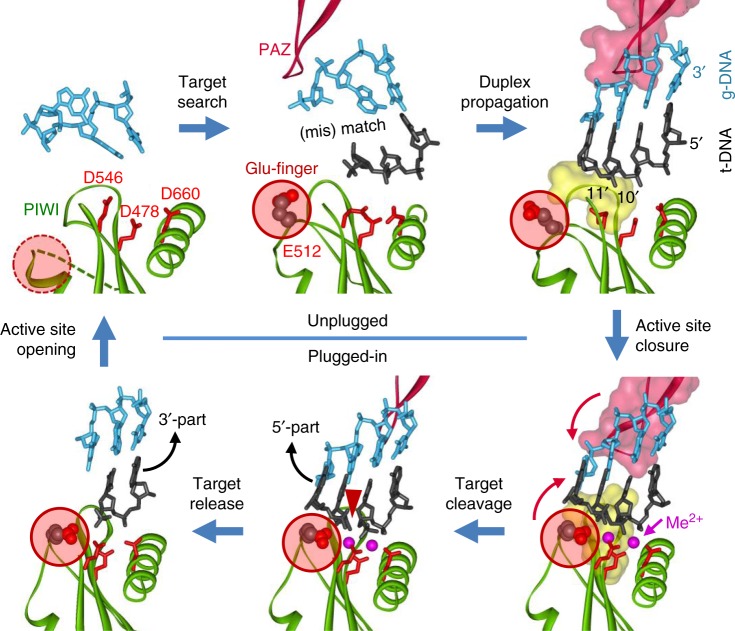
Conformational changes in the active site of TtAgo during target recognition and catalysis^29,30^. The active site residues are shown in red; the glutamate finger is indicated with a red circle. During first steps of guide binding and target recognition, the active site is unplugged (upper raw); duplex propagation is accompanied by changes in the conformations of the PIWI and PAZ domains (indicated with red arrows), plugging-in of the glutamate finger, catalytic metal binding, and activation of catalysis (lower raw). Finally, stepwise target release leads to unplugging of the active site, thus making possible recognition of the next target molecule. The PDB accession numbers (from the upper left corner, clockwise): 3DLH, 3F73, 4N41, 4NCB, 4NCA, 4N76 (see Fig. 2 and Supplementary Fig. 2)

The binding of complementary nucleic acid target is accompanied by structural changes of the Ago molecule that include rotations of the PAZ domain and changes in the conformations of several loops in the PIWI domain, resulting in closure of the nucleic acid duplex within the catalytic cleft of pAgo and activation of catalysis, as described below (Figs. 2,  3)^27,29,30,36,37^.

The catalytic site of all active Ago proteins contains a conserved tetrad of negatively charged amino acid residues, DEDX (where X is D, H, or K) that chelate catalytic divalent metal ions, Mg^2+^ or Mn^2+^ (Fig. 3)^9,10^. Mn^2+^ usually increases pAgo activity, and some pAgos (PfAgo) were shown to be active only in the presence of manganese ions^26,29,31,36^. The catalytic glutamate residue is located in the so-called glutamic finger that can adopt different conformations. In the absence of a target, it is located away from the catalytic site (“unplugged”), the complete tetrad is not formed, and no metal ions are bound in the active site (or only a single ion is observed) (Fig. 3, Supplementary Fig. 2; Supplementary Table 1)^22,28,36^. Formation of the extended guide-target duplex is accompanied by its closure within the nucleic acid binding cleft of pAgo, due to conformational changes in the PIWI and PAZ domains (indicated with red arrows in Fig. 3), insertion of the glutamic residue into the active site (“plugged in” conformation), binding of catalytic metal ions and activation of target cleavage (Fig. 3 and Supplementary Fig. 2)^29,30^.

Catalytically inactive pAgos, such as RsAgo, contain substitutions of one or more negatively charged residues in the active site^9,10^. In addition, RsAgo remains in the unplugged conformation even after ternary complex formation, which also prevents catalytic metal binding (Supplementary Fig. 3)^35,51^. In contrast to pAgos, the catalytic site of the AGO-clade eAgos (hAgo2, hAgo3, KpAgo) was always found in the “plugged in” conformation, independently of the guide and target binding (Supplementary Fig. 3)^38,40,43,53,54^. At the same time, the PIWI-clade SIWI protein adopted the unplugged conformation in the absence of a target, suggesting that it may be more closely related to pAgos^44,58^ (see below).

For most studied catalytically active Agos, the target is cleaved precisely between positions complementary to the 10th and 11th nucleotides of the guide strand (Figs. 2,  3)^18,22,23,27,29,36^. Intriguingly, more than one cleavage site was observed for MjAgo^21–23^ but the structural basis for this remains unknown. Analysis of catalytically active eukaryotic and prokaryotic Ago proteins demonstrated that they are multiple turnover enzymes. Target release was shown to be the rate-limiting step in the action of eAgo proteins, due to persisting complementary guide-target interactions after target cleavage^25,48,49,59,60^. Mismatches in both the seed region and the 3′-supplementary guide site increase the enzyme turnover, although at the cost of decreased target binding^48,49,60^. At the same time, target release is not rate limiting for catalysis by the thermophilic TtAgo protein^29^, for which the high temperature used in the assays likely promotes target dissociation. It remains to be established whether other protein factors may assist target release for pAgos from mesophilic prokaryotes.

A structural insight into the process of target release was obtained from the analysis of a ternary complex of TtAgo that was incubated at high temperature after target cleavage before crystallization (Figs. 2,  3, Supplementary Fig. 2)^30^. As revealed in the structure, the cleaved 5′-part of the target strand has dissociated from TtAgo and the corresponding 3′-portion of the guide is disordered (Fig. 3, bottom left). FRET measurements demonstrated that dynamic 3′-guide re-association with the PAZ pocket likely promotes target release^56^. This is likely followed by dissociation of the 3′-part of the target strand and unplugging of the active site, thus regenerating the binary guide-pAgo complex for the next round of catalysis. Analysis of eukaryotic Agos revealed the same sequential pathway of target dissociation, which can change depending on the presence of mismatches in the seed and 3′-supplementary guide sites^48,49^.

### Recognition of mismatched vs. matched targets

In eukaryotes, the efficiency of target repression by Ago-containing effector complexes greatly depends on the extent of complementarity between the guide and target RNAs^61–67^. Although a possible functional importance of the mismatched target recognition by pAgos remains unknown (in the context of their cellular functions discussed below), their further analysis may shed light onto the mechanisms of target recognition and various silencing pathways in both prokaryotes and eukaryotes.

**Fig. 4:**
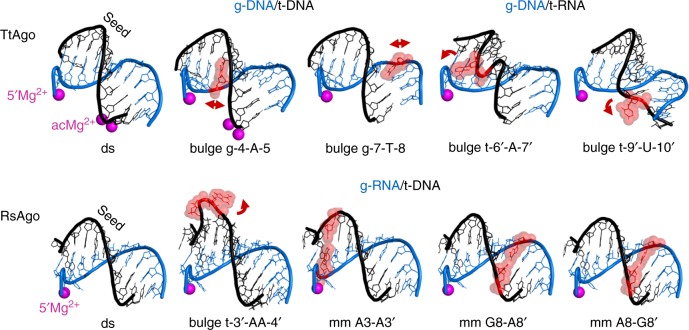
Accommodation of helical imperfections in the ternary complexes of pAgo proteins. Structural features of the duplexes formed in the seed region in ternary complexes of TtAgo^71^ (upper raw) and RsAgo^51^ (bottom) containing bulges or mismatches (shown in red) in the guide or target strand, in comparison with fully double-stranded duplex (“ds”). Only the part of the duplex between the guide 5′-end and the active site in the PIWI domain is shown (guide positions 1 through 10–12 for various complexes); Mg^2+^ ions bound in the MID-pocket (5′Mg^2+^) and in the active site (acMg^2+^) are indicated; some complexes of TtAgo were obtained with a catalytically inactive mutant and thus lack catalytic metal ions. The distortions of the double-helix are shown with red arrowheads; the nucleotide bulges can be either stacked-in (bulges in the guide strand; g-4-A-5 and g-7-T-8 in TtAgo) or flipped-out of the duplex (bulges in the target strand; t-6′-A-7′, t-9′-U-10′ for TtAgo, t-3′-AA-4′ for RsAgo). The ternary complexes were obtained with g-DNA/t-DNA or g-DNA/t-RNA for TtAgo, or g-RNA/t-DNA for RsAgo, as indicated. The PDB accession numbers are (from left to right): TtAgo, 4NCB, 5XP8, 5XOU, 5XOW, 5XPA; RsAgo, 6D8P, 6D8A, 6D92, 6D9L, 6D9K. See Supplementary Table 1 and Supplementary Fig. 3 for full description of each complex

Mismatches in the seed region between miRNAs and siRNAs and their targets have the most deleterious effects on the efficiency of silencing in eukaryotes^48–50,68–70^. Similarly, mismatches and bulges within the seed region significantly impair target binding and cleavage by studied pAgos^17,27,37,51,71^. No information on the structure of mismatched complexes is available for eAgo proteins. However, recent studies unexpectedly revealed that TtAgo and RsAgo can accommodate helical imperfections within the seed region in ternary complexes with only moderate structural perturbations (Fig. 4 and Supplementary Fig. 4)^51,71^. It was shown that purine-purine mismatches in the seed region can be bound without significant distortions of the duplex (e.g., mm A3-A3′, mm G8-A8′, mm A8-G8′ for RsAgo, Fig. 4). Nucleotide bulges in the guide strand in the ternary complexes of TtAgo stack-in between adjacent bases resulting in local distortions of the double helix (e.g., bulges g-4-A-5 and g-7-T-8, Fig. 4). In contrast, bulges in the target strand, which is more solvent-exposed, were shown to be looped-out of the duplex (e.g., bulges t-6′-A-7′ and t-9′-U-10′ for TtAgo, t-3′-AA-4′ for RsAgo, Fig. 4), resulting in stronger helix distortion and, in some cases, shifting of the cleavage site^51,71^.

Intriguingly, the presence of bulges or mismatches in the seed region was shown to stimulate release of the imperfect guide-target hybrid from RsAgo, thus providing a mechanism for rapid guide exchange and Ago recycling^51^. Similarly, it was recently shown that mismatches in the seed region promote unloading of miRNAs from human Ago2, suggesting that such mechanism of guide exchange may be conserved in evolution^72^.

Mismatches and bulges around the active site greatly decrease the efficiency of target cleavage by most studied eAgos^48,49,68,70^ and pAgos alike^27,37,71^. From the structural perspective, mismatches at the cleavage site disrupt protein-nucleic acid interactions in the ternary complexes of TtAgo (in some mismatched complexes, the downstream part of the duplex is completely disordered) and the active site remains in the open unplugged conformation (Fig. 3, Supplementary Figs. 2 and 4)^27,29,71^. Thus, formation of the perfect guide-target duplex in the active site is a critical checkpoint in the specific target cleavage by Ago proteins, and the presence of helical imperfections hampers structural transitions required for activation of catalysis.

## Functional activities of pAgos

It was initially proposed that pAgos might provide defense against foreign genetic elements such as transposons, phages and plasmids^9^. This hypothesis has found experimental support in recent studies of two long pAgos, catalytically active TtAgo and inactive RsAgo. The properties of these two proteins were most extensively studied in vitro and in vivo thus making them favorable models to understand functional activities of pAgos.

### DNA-guided interference by TtAgo

TtAgo is an active endonuclease that binds DNA guides to cleave complementary DNA or RNA targets in vitro^27,29–31^. When purified from bacterial cells, TtAgo is associated exclusively with short DNA molecules^31^. The preferable substrate for TtAgo in vitro is ssDNA but it can also cleave plasmid substrates, when provided with guide molecules complementary to the two DNA strands^31^. The plasmid cleavage depends on DNA supercoiling or the presence of A/T-rich regions and occurs only at elevated temperatures, suggesting that it requires local DNA melting^31,33^. Deletion of TtAgo from the genome of *T. thermophilus* increases the efficiency of natural transformation and plasmid yield suggesting that TtAgo can also target plasmid DNA in vivo^31^.

One of the most intriguing questions is how target-specific DNA guides associated with TtAgo and other DNA-loaded pAgos are generated. Cloning and sequencing of small DNAs (13–25 nucleotides in size) associated with TtAgo during expression in a heterologous *E. coli* system revealed that they predominantly originate from plasmids and are uniformly distributed over replicons, independently of the G/C-richness, gene content and orientation^31^. Importantly, these small DNAs were absent upon expression of a mutant TtAgo with substitutions of catalytic residues in the active site indicating that guide DNA formation depends on its catalytic activity.

Small DNA molecules associated with TtAgo in vivo have a strong preference for cytosine at their 5′-end (g1C)^31^ but in vitro analysis demonstrated that TtAgo rather recognizes complementary guanosine residue in the target DNA strand (t1G′). This suggests that initial substrate for TtAgo is dsDNA and that selection of 5′-C-containing guides occurs during guide loading prior to removal of the complementary strand^33^. Indeed, prolonged incubation of guide-free TtAgo with double-stranded substrates, but not ssDNA, resulted in their cleavage^33^. This activity, termed DNA ‘chopping’, required the presence of A/T-rich or mismatched DNA regions, preferably located in the 5′-direction relative to the site of cleavage.

Other studied DNA-guided pAgo proteins revealed similar activities in vitro (Table 1). Thermophilic AaAgo, MjAgo and PfAgo exhibited efficient guide-dependent cleavage of single-stranded or supercoiled plasmid substrates^18,22,23,26^. At elevated temperatures (≥75 °C), MjAgo and PfAgo also cleaved linear or plasmid double-stranded DNA substrates without the addition of guide molecules^23,26^. Although hyperthermophiles (such as *P. furiosis* and *M. piezophila*) usually contain reverse gyrase to positively supercoil their DNA, the extreme temperatures of their habitats likely promote local DNA melting. Thus, catalytically active pAgos can autonomously initiate DNA cleavage and produce specific guide molecules for the same target, and may not require additional factors for initiation of DNA interference in vivo.

**Fig. 5:**
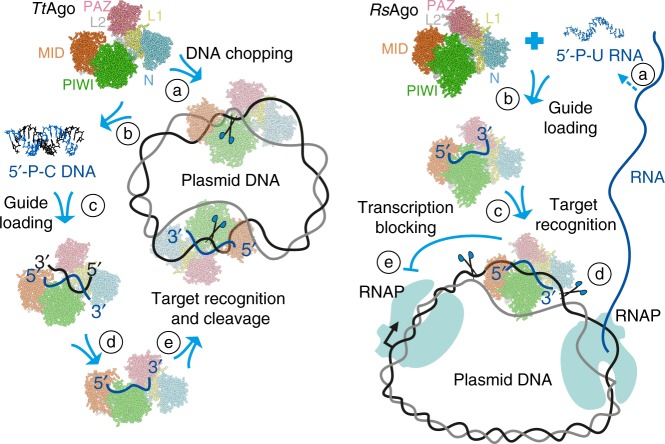
Proposed mechanisms of DNA interference by DNA-guided (TtAgo, left) and RNA-guided (RsAgo, right) pAgos. TtAgo was proposed firstly to process invader DNA in a guide-independent manner (“DNA chopping”, a), resulting in slow DNA fragmentation (b) and binding of short DNA duplexes (c), followed by dissociation of the passenger strand (d). Guide-loaded TtAgo can then attack the target DNA with high efficiency (e)^31,33^. RsAgo was proposed to bind short RNAs processed from mRNAs by Ago-associated or cellular nucleases (a, b), followed by target DNA recognition (c), which can result in DNA degradation by accessory nucleases (d) and/or inhibition of transcription (e)^34^

These studies have led to the model of specific DNA targeting by TtAgo and other DNA-guided pAgos schematically shown in Fig. 5 ^31,33^. Guide-free TtAgo initially attacks double-stranded DNA substrates (step a) and makes distributed nicks on each DNA strand, thus resulting in generation of double-stranded fragments of varying length (step b). This is a low-efficiency process that may be stimulated by the presence of partially single-stranded regions or noncanonical DNA structures. Next, guide molecules are selected from the pool of these fragments based on the presence of guanine in the passenger strand opposite first guide cytosine (step c), whose binding in separate protein pockets may facilitate strand separation. This is followed by dissociation of the passenger strand, either with or without its cleavage, stimulated by the presence of an A/T-rich segment in its 5′-part (step d). Guide-loaded TtAgo then attacks the same DNA target with high efficiency and specificity, resulting in the decrease in plasmid transcription and its further degradation (step e).

### RNA-guided interference by RsAgo

RsAgo uses RNA guides to recognize complementary DNA targets in vitro but lacks the slicer activity due to substitutions of key catalytic resides in the active site (Supplementary Fig. 3)^34,35^. However, when purified from the host cells, RsAgo is associated with small 15–19 nt RNA and complementary 20–25 nt DNA molecules of diverse sequences^34^. The RsAgo-bound guide RNAs contain a 5′-uridine residue (gU1) and complementary DNAs have an adenine at corresponding position (tA1′) close to their 3′-end^34^; these residues are specifically recognized by RsAgo in vitro^35,51^.

Small RsAgo-associated RNAs correspond to the sense strand of the genes suggesting that they are processed from cellular RNA transcripts. Little gene specificity was observed for these RNAs, though moderate enrichment for plasmid-derived and transposon transcripts, and depletion of noncoding RNAs was reported^34^. In the *R. sphaeroides* genome, RsAgo is located in the same operon with a downstream gene encoding putative nuclease. However, RsAgo still associates with small RNAs and DNAs when expressed without nuclease either in *R. sphaeroides* or in *E. coli* cells suggesting that the nuclease is not essential for nucleic acid processing and RsAgo may “collect” short RNAs from the pool of cellular RNAs processed by various RNases.

In *R. sphaeroides*, RsAgo decreases the expression of plasmid genes without obvious plasmid degradation^34^. When expressed at high levels in *E. coli*, it also decreases plasmid content and causes plasmid degradation, suggesting that it can affect not only transcription but also DNA integrity^34,35^. The mechanism of DNA processing remains unknown; however, since RsAgo lacks catalytic activity and small DNAs are processed outside of the region of complementarity to guide RNAs, the involvement of other cellular DNases was proposed^34^. An even bigger mystery is the observed specificity of target DNA recognition, since despite promiscuous association of RsAgo with RNA guides, the complex seems to target foreign DNA, particularly transposons, plasmids and prophages^34^.

Overall, these studies suggested the model of RNA-guided interference by RsAgo shown in Fig. 5^34^. Initial processing of RNA transcripts by cellular nucleases results in generation of a pool of RNA fragments corresponding to both host and foreign genes (step a). Guide molecules are selected by RsAgo from this pool by their size and the presence of 5′-uridine, probably followed by the 3′-end trimming (step b). At this stage, certain properties of foreign RNA transcripts, such as low efficiency of translation, may distinguish them from host protein-coding genes (which have optimal expression patterns) or structured noncoding RNAs (protected from degradation), thus allowing preferable guide loading. At the next step, the RsAgo-RNA complex binds target DNA of corresponding genetic loci (step c). This process may be facilitated by gene transcription, which promotes local negative DNA supercoiling and melting behind RNA polymerase^73^. The presence of bound pAgo may directly affect gene transcription, by imposing a roadblock to RNA polymerase (step d). Finally, DNA-bound RsAgo complexes can be removed from the genome by the action of unknown nucleases, resulting in the appearance of single-stranded gaps and double-stranded breaks in the DNA target (step e). Similarly to DNA-guided pAgos, this may lead to degradation of the target replicons.

## Commonalities and differences in the action of pAgo and eAgo proteins

At the molecular level, prokaryotic and eukaryotic Argonaute proteins are strikingly similar in the mechanisms of nucleic acid binding and slicer activity, suggesting that the basic function of Argonautes is conserved in evolution^10,11^, but with certain variations discussed below. In eukaryotes, Ago proteins have evolved to use RNA guides (siRNA and miRNA) to regulate gene expression at post-transcriptional level through recognition of RNA targets in the cytoplasm. In addition, nuclear Ago proteins in fission yeast and plants as well as nuclear PIWI-clade Agronautes in Metazoa induce transcriptional repression through binding to nascent RNAs in the nucleus^74–78^. In contrast, most studied pAgos, including archaeal proteins that likely served as predecessors of eAgos^10,11^, use DNA guides to recognize DNA targets. Yet some pAgos like RsAgo and MpAgo utilize RNA guides, and it is not unlikely that RNA-targeting pAgos may also be discovered in the future, similarly to RNA-targeting CRISPR-Cas systems^79^. In fact, several pAgos including AaAgo, TtAgo and MpAgo, were shown to cleave RNA targets in vitro, although usually with lower activities in comparison with DNA targets^18,27,36,80^. The functional role of this activity in vivo remains to be established.

In contrast to eAgos, which require accessory proteins for guide generation and loading, small DNA or RNA guide loading into pAgos does not seem to depend on the action of additional proteins. Both TtAgo and RsAgo successfully associate with small nucleic acids in heterologous bacteria species^31,34^, and initial DNA processing and guide loading by TtAgo and MjAgo in vitro does not require any accessory factors^23,33^. No chopping activity was reported for eAgos, but some specific miRNAs and synthetic siRNAs can be processed by the slicer activity of the Ago2 protein, without the need for Dicer, in a certain analogy with pAgos^61–63,81,82^. However, as shown for RsAgo, the mechanism of RNA-guided repression in prokaryotes is conceptually very different from RNAi in eukaryotes: while in eukaryotes guide RNAs are carefully selected to achieve the specificity of target recognition, in prokaryotes the selection is not driven simply by RNA guides and occurs—by as yet unknown mechanism—at the step of target (in this case, DNA) recognition by the guide-pAgo complex^34^. A specific group of RNA-guided CRISPR-associated pAgos, such as MpAgo, might use cellular memories of previous infections encoded in the CRISPR cassette for the recognition of foreign nucleic acids, but this has not been demonstrated experimentally yet^36,37^.

The double-stranded nature of DNA implies that it should be premelted for guide-dependent recognition by pAgos, in contrast to eAgos that act on single-stranded RNA targets. DNA targeting seems to be a straightforward mechanism of gene silencing in prokaryotes, but may become inefficient in the case of eukaryotic cells, in which genomic DNA is tightly packed into chromatin, while gene activity is also highly regulated at post-transcriptional level—thus explaining the switch of eAgos to the RNA silencing activity. Indeed, DNA chromatinization was proposed to protect the genome (but not invader DNA) from the action of MjAgo in the archaeon *M. jannaschii*^23^. At the same time, some eAgos were proposed to recognize DNA in vivo (*A. thaliana* AGO4 and AGO1, mammalian Ago2)^83–85^ and can use DNA guides for target recognition in vitro (hAgo2)^42^, suggesting that their ability to interact with DNA might not be lost in evolution.

Suppression of foreign genetic elements by pAgos parallels the functions of the PIWI-clade eAgos and piRNAs in transposon silencing^86–89^. Furthermore, pAgos may possibly suppress gene expression at the transcriptional level^34^, analogously to the piRNA pathway in eukaryotes^64–67,90,91^ (see next section). Recent analysis of the SIWI protein from the PIWI clade revealed structural similarities with pAgos, including the unplugged conformation of the active site and the metal-mediated 5′-guide interactions in the MID pocket. PIWI proteins may therefore represent an ancient functional variant of eAgos^44,58^.

## Possible cellular functions of pAgos

**Fig. 6:**
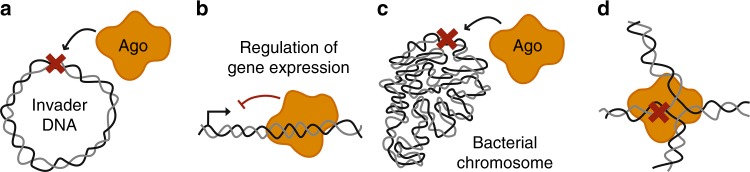
Possible functions of pAgos. In addition to their function in cell defense against invader DNA (or RNA) (**a**), pAgo proteins might hypothetically be involved in the regulation of gene expression (**b**), function as suicide systems (**c**), or participate in the processing of noncanonical DNA structures and DNA repair (**d**)

While published studies proposed that elimination of foreign genetic elements through their nucleolytic cleavage may be the main mode of action for pAgos (Figs. 5,  6a; Table 1), we hypothesize that these proteins might also be implicated in the regulation of other genetic processes, not necessarily requiring DNA cleavage.

RNA-guided pAgos that lack endonuclease activity, such as RsAgo, may perform cleavage-independent repression of foreign genes (Fig. 6b). Indeed, repression of plasmid-encoded genes was observed in *R. sphaeroides* strains expressing wild-type RsAgo, without changes in the plasmid copy-number^34^. Small DNAs found in association with RsAgo in vivo^34^ may possibly be a byproduct of RsAgo binding to specific DNA loci with occasional DNA cleavage, while its main function might be in transcriptional silencing. In particular, RsAgo could co-transcriptionally bind its genomic targets, thus preventing next rounds of transcription (Fig. 5)^34^. We hypothesize that such inhibition may be more efficient for foreign genes because of their inefficient translation, which is associated with lower speed of transcription and RNA polymerase backtracking^92^. Intriguingly, recent studies suggested that, similarly to RsAgo, the plant AGO4 protein may directly recognize DNA targets and prevent their further transcription through heterochromatinization^83^.

Prokaryotic Ago proteins may also be involved in transcriptional regulation of host genes. In the case of eukaryotes, transcriptional repression is achieved through recognition of nascent RNA by a complex of nuclear eAgo and small RNA, followed by recruitment of chromatin modifiers that put repressive chromatin marks on the target locus^66,91,93–95^. Nuclear eAgos induce transcriptional silencing in fission yeast and plants, while the PIWI-clade Argonautes and associated piRNAs are responsible for transcriptional silencing of transposable elements in germ cells of Metazoa. In contrast to eAgos that bind nascent RNAs, loading of pAgos onto genomic loci in prokaryotic cell may directly interfere with gene transcription, similarly to DNA-binding transcription repressors (Fig. 6b). At present, no studies of the effects of RNA-guided pAgos on the expression of chromosomal genes were reported, but RsAgo was shown to repress transcription of plasmid genes^34^. Intriguingly, TtAgo stimulates (directly or indirectly) expression of certain chromosomal genes, including the CRISPR-Cas locus, in *T. thermophilus* strains containing plasmid DNA, suggesting a functional interplay between the pAgo and CRISPR systems^32^. Efficient transcription inhibition in bacterial cells was previously reported for a catalytically inactive variant of the Cas9 nuclease loaded with gene-specific RNA guides^96^. It will be important to explore if pAgos might also be adopted for synthetic regulation of gene expression.

Beyond repression of foreign genetic elements and host genes, pAgos might act as a suicide system similar to abortive infection systems (reviewed in ref. ^97^) that kill a bacterial cell under stress conditions (Fig. 6c). A similar function was also proposed for CRISPR-Cas systems^98,99^. In this scenario, environmental stress, extensive DNA repair or phage infection result in the appearance of partially melted DNA regions, which may be a preferable substrate for pAgo action, resulting in pAgo loading with small DNA fragments corresponding to genomic sequences. ssDNA-guided pAgos can then effectively destroy DNA, thus resulting in cell death and preventing phage multiplication.

Finally, we hypothesize that pAgos might act as components of an ancient DNA repair pathway, by inducing DNA cleavage at the sites of noncanonical DNA structures, such as broken replication forks, 5′-flaps, Holliday junctions, and R-loops (Fig. 6d). Previously, a DNA repair function was proposed for CRISPR-Cas systems^100^, and CRISPR-associated nucleases have indeed been shown to play various roles beyond interference (reviewed in ref. ^99,101^). In particular, the Cas1 protein from *E. coli* can process a variety of noncanonical DNA substrates in vitro^102^, beyond the canonical DNA integration intermediates recognized by the Cas1-Cas2 complex^103,104^. Cas1 also physically and genetically interacts with DNA recombination factors in vivo, and its deletion renders the cells more sensitive to DNA damage^102^. Furthermore, the CRISPR-system was shown to attack noncanonical DNA substrates—mostly, damaged replication forks—and cooperate with cellular DNA repair pathways during spacer acquisition^105^. Recently, partially complementary regions were shown to promote guide-independent DNA cleavage by TtAgo^33^. Thus, we speculate that the nuclease activity of pAgos towards unusual DNA structures might stimulate their processing by other cellular nucleases and repair proteins.

eAgo proteins have been implicated in double-strand break (DSB) repair in plant and human cells, in a process that requires transcription^85,106,107^. Small RNA-loaded Ago2 was proposed to recognize the sites of DSBs through pairing with complementary DNA sequences or nascent RNA transcripts, followed by recruitment of other DSB repair proteins^85^. Moreover, Ago1 in plants was shown to interact with DNA damage-binding protein 2 (DDB2) and, possibly, facilitate recognition of the sites of UV-damage through direct base-pairing with the DNA substrate^84^. Stress-induced DNA targeting by pAgos, possibly coupled to transcription, might also play a role in DNA repair and in stress response in prokaryotic cells.

## Future directions in pAgo studies

Many functional features of the proposed bacterial DNA/RNA interference systems, as well as possible regulatory pathways involving pAgos, remain to be established. The experimental evidence for their role in host defense is still very limited; for example, nothing is known about their possible effects on the replication of bacteriophages, the most abundant bacteria-targeting genetic elements. The three principal questions that have to be answered about pAgos are (1) how the nucleic acid guides associated with pAgos are generated, (2) what are the natural targets of the pAgo/guide complexes and how are they selected, and (3) what happens with the target upon its recognition by these complexes. Some specific problems that need to be addressed about pAgos are briefly outlined below.

### Guide biogenesis

The molecular pathways of guide biogenesis are certainly different for RNA-guided and DNA-guided pAgos, and it remains to be known how the nucleic acid substrates are selected for initial processing. While DNA chopping was shown to be a route for guide generation in vitro^23,33^, not all pAgos show this activity, and it still remains a question how the nucleic acid guides are generated in vivo. Since DNA chopping requires DNA premelting^33^, partially single-stranded DNA that appears during invasion and replication of mobile genetic elements might be first attacked by non-guided pAgos. In the case of CRISPR/Cas systems, the RecBCD exonuclease was shown to process DNA for spacer generation during the adaptation step of CRISPR/Cas-interference^105^. The same system might contribute to preferable processing of foreign DNA into DNA guides utilized by pAgo proteins.

The RNA guide biogenesis may depend on the transcription-translation coupling (not existing in eukaryotes), which may drive RNA processing and guide loading into pAgos. The features that might make an mRNA a preferable source of guide molecules include its inefficient translation (which makes RNA unprotected by the ribosomes)^74^, or specific secondary structure. The nucleases involved in RNA cleavage are unknown but likely candidates include pAgo-associated proteins encoded in the same operons. It remains to be known whether Cas nucleases may participate in guide RNA processing in the specific case of CRISPR-Cas-associated pAgos (MpAgo)^36^. It will be also interesting to test whether pAgos can also perform guide-independent cleavage of (partially double-stranded) RNA precursors, similarly to the processing of a subclass of miRNAs by eAgo2^61,62,81^.

### Target selection

Almost nothing is known about the mechanisms that may target pAgos to specific genomic loci or foreign replicons, such as extrachromosomal DNA, transposons, plasmids or phages. Unusual replication properties of these elements can lead to the formation of partially single-stranded DNA intermediates that may be preferably recognized by guide-loaded pAgos^31,33,34^. Single-stranded DNA regions can appear in the cell during DNA repair and transposition, or as a result of perturbed transcription. Single-stranded DNA can also enter the cell during the processes of conjugation and natural transformation, thus making horizontally acquired DNA more susceptible to the pAgo action. The multicopy nature of plasmids and transposable elements can rise the number of produced guide molecules and may induce silencing when this number exceeds a threshold level. For MjAgo, DNA coverage by archaeal histone proteins was proposed to protect genomic DNA from cleavage thus making plasmids more susceptible for Ago action^23^. Architectural DNA binding proteins may introduce a similar bias in bacteria.

Gene-specific differences in the transcription and translation levels may also affect target selection. In prokaryotes, foreign DNA sequences are less efficiently translated because of suboptimal codon bias^74^. Decreased translation results in lower rates of transcription due to inefficient transcription-translation coupling and increased RNA polymerase backtracking^92^, which may in turn affect DNA replication and repair^75,108^, and co-transcriptional pAgo loading.

### Target processing

The mechanisms of target degradation by pAgos in vivo remain poorly understood. For TtAgo, short DNAs are uniformly distributed along a target plasmid, arguing against sequence-dependent or ordered DNA cleavage^31^; nothing is known about in vivo DNA processing by other catalytically active pAgos. It is plausible that other cellular nucleases, such as homologous recombination machinery, may contribute to dsDNA processing (similarly to the CRISPR-Cas interference^105^). The RecBCD system might participate in plasmid degradation after its initial cleavage by pAgo proteins, resulting in its preferable processing resulting from the absence of Chi-sites. Recently, it was shown that in vitro cleavage of double-stranded DNA by TtAgo can also be promoted by the UvrD helicase and the SSB protein^109^; however, it remains to be established whether these or other factors also facilitate DNA processing in vivo.

Catalytically inactive pAgos, such as RsAgo, process target DNA by an unknown mechanism that may involve the action of pAgo-associated nucleases. Furthermore, it remains unknown whether DNA cleavage is an essential step in the action of these type of pAgos, since their strong association with DNA may by itself affect target replication, transcription and repair, as discussed above^34^.

### Functional activities of short pAgos

While short pAgos constitute a large part of all pAgos, their functional activities and the ability to interact with nucleic acids in vivo were never tested (and hence their DNA/RNA specificity remains unknown). Short pAgos lack the N-terminal half of the protein, including the PAZ and MID domains involved in guide binding and target recognition (Fig. 1, AfAgo), and contain inactivated catalytic site. Furthermore, the path of DNA and RNA duplexes bound by AfAgo in reported structures (Fig. 1) significantly differs from long pAgos, suggesting that other (APAZ-containing) proteins encoded in the same operons may participate in DNA/RNA binding and processing.

### Noncanonical pAgo functions

As we argue in this review, protection against invader DNA may not be the only cellular function of pAgo proteins (Fig. 6). To date, detailed in vivo studies have been performed for only two proteins (TtAgo and RsAgo) from the highly divergent evolutionary tree of pAgos. The detailed understanding of possible pAgo roles in genetic regulation, stress response and DNA repair will therefore require study of new bacterial and archaeal pAgos, selected on the basis of their evolutionary and functional diversity^10,11^, and the availability of convenient genetic systems for their analysis.

### The use of pAgos in genetic engineering

In addition to understanding pAgo function in their host prokaryotic cells, it is worth exploring the possibility to use pAgos as tools for transcription regulation, genome editing and epigenome rewriting^13^. Initial attempts to use an archaeal Ago protein for genome editing were irreproducible^110–112^ but analysis of diverse pAgos found in various bacterial and archaeal species may help to select better candidates for genome manipulations. Further studies may help to find efficient RNA-targeting pAgos, which, in contrast to eAgos, will not interfere with the cellular RNAi pathways. Several studied pAgos (AaAgo, MpAgo, TtAgo) are able recognize and cleave RNA in vitro^18,27,36,80^, and MpAgo was recently adopted for detection of specific RNA species from complex mixtures^80^. The main problems that need to be solved include the directing of pAgos to desired genomic locations or mRNA targets and avoiding off-target effects. For this purpose, pAgos can be fused with additional domains for specific loading of RNA or DNA guides and chromatin modification^110^.

## Electronic supplementary material

Supplementary Information

## Data Availability

No datasets were generated or analysed during the current study.
