# Supplementary material for: DNA interference and beyond: structure and functions of prokaryotic Argonaute proteins
Source: Nat Commun. 2018 Dec 4;9:5165. doi: 10.1038/s41467-018-07449-7 (PMC6279821; doi:10.1038/s41467-018-07449-7)
Supplement: Supplementary file 1 — Supplementary Information [file 41467_2018_7449_MOESM1_ESM.pdf]

DNA interference and beyond:

structure and functions of prokaryotic Argonaute proteins

Lidiya Lisitskaya[1], Alexei A. Aravin[1,2]*, Andrey Kulbachinskiy[1]*

[1] Institute of Molecular Genetics, Russian Academy of Sciences, Moscow 123182, Russia

[2] Division of Biology and Biological Engineering, California Institute of Technology, Pasadena, CA 91125, USA

Supplementary information

This document contains four Supplementary Figures 1-4 and Supplementary Table 1.

Supplementary Fig. 1. Comparison of known structures of pAgo (a) and eAgo (b) proteins. *(next page)*

The structural elements are colored as follows: N-domain, turquoise; L1, yellow; PAZ, magenta; L2, grey; MID, orange; PIWI, green. The guide strand is blue, the target strand is black. Metal ions bound in the MID-pocket (5′Me^{2+}) or in the active centre ($acMe^{2+}$) are indicated. Arrows indicate major changes in the PAZ domain orientation. The PDB accession numbers are shown next to each structure.

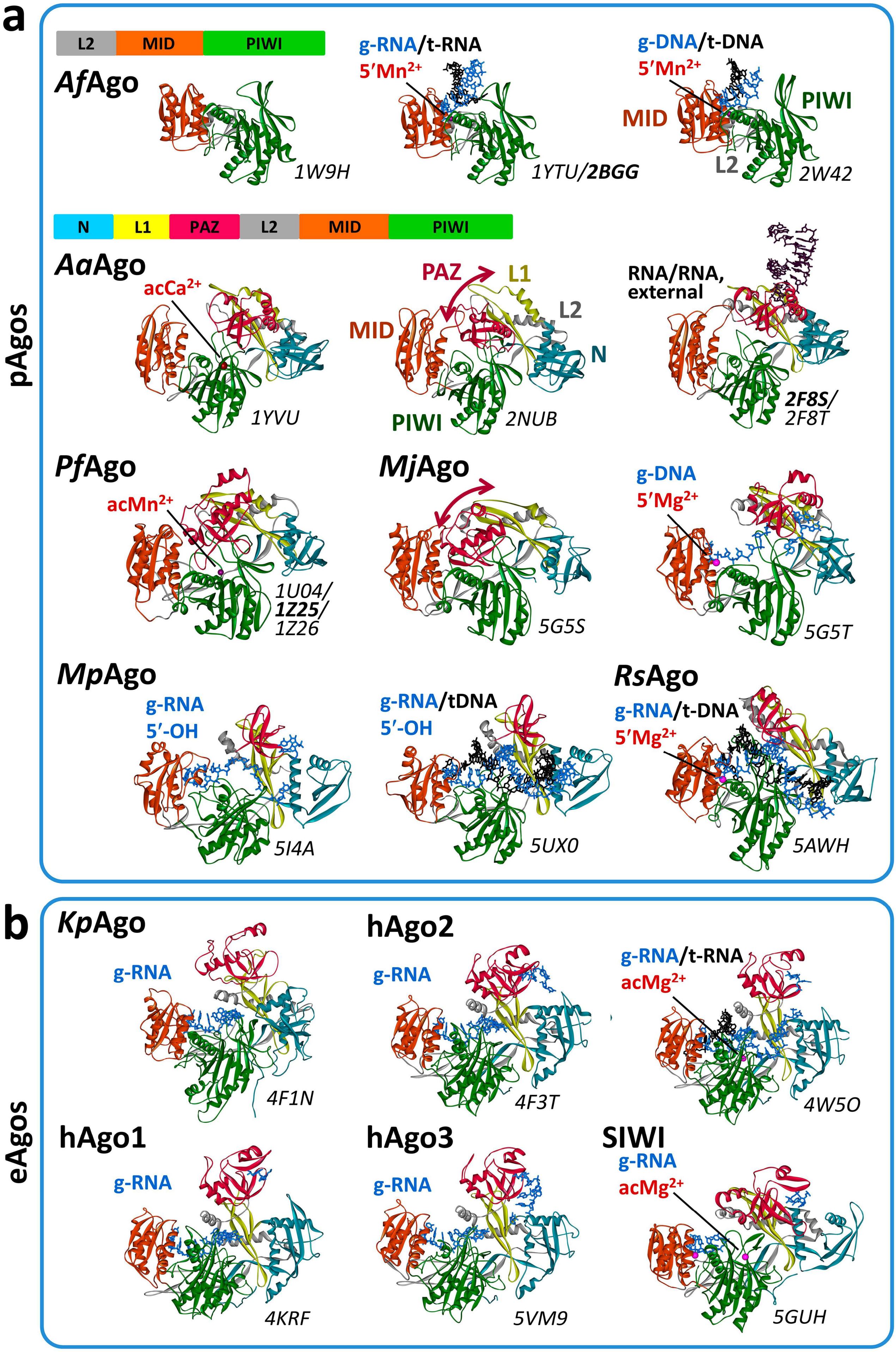
a
L2
MID
PIWI
AfAgo
1W9H
g-RNA/t-RNA
5′Mn2+
1YTU/2BGG
g-DNA/t-DNA
5′Mn2+
MID
PIWI
L2
2W42
N
L1
PAZ
L2
MID
PIWI
AaAgo
acCa2+
1YVU
PAZ
L1
L2
MID
N
PIWI
2NUB
RNA/RNA, external
2F8S/
2F8T
PfAgo
acMn2+
1U04/
1Z25/
1Z26
MjAgo
5G5S
g-DNA
5′Mg2+
5G5T
MpAgo
g-RNA
5′-OH
5I4A
g-RNA/tDNA
5′-OH
5UX0
RsAgo
g-RNA/t-DNA
5′Mg2+
5AWH
pAgos
b
KpAgo
g-RNA
4F1N
hAgo2
g-RNA
4F3T
g-RNA/t-RNA
acMg2+
4W5O
hAgo1
g-RNA
4KRF
hAgo3
g-RNA
5VM9
SIWI
g-RNA
acMg2+
5GUH
eAgos

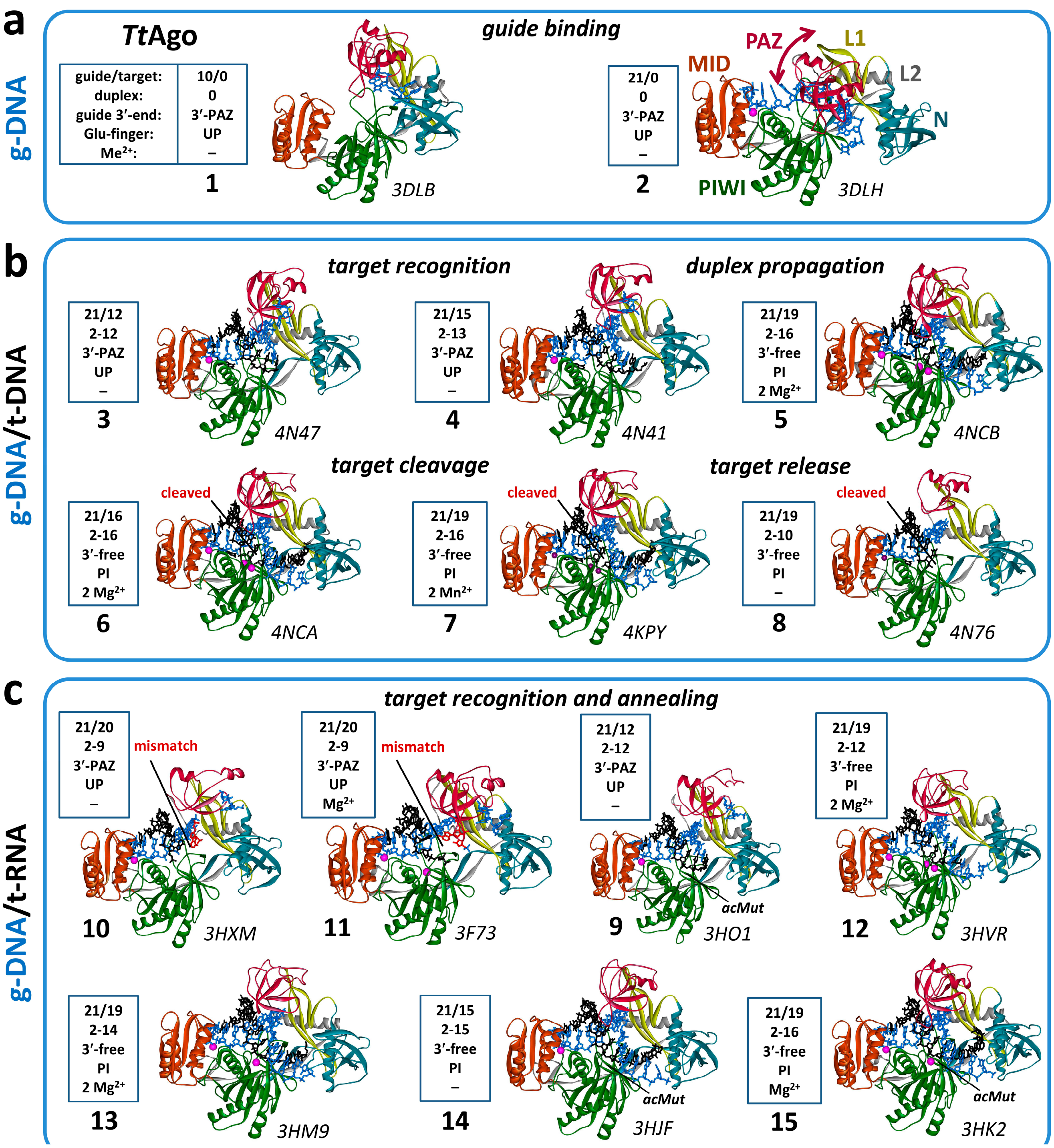

Supplementary Fig. 2. Structures of the TtAgo complexes at different steps of the catalytic cycle. (a) Binary complexes with guide DNA (1-2) [1]. (b) Ternary complexes with guide DNA and target DNA (3-8) [2]. The numbering of the structures corresponds to consecutive steps of the catalytic cycle of TtAgo: 1-2 – g-DNA binding; 3-4 – t-DNA recognition; 5 – duplex propagation and catalytic Me^{2+} binding; 6-7 – t-DNA cleavage with Mg^{2+} and Mn^{2+} ions; 8 – t-DNA release (crystallized after heating the complex at 55°C). (c) Ternary complexes with guide DNA and target RNA (9-15) with increasing lengths of the duplex [3, 4]. Complexes 9 and 10 contain mismatches at guide positions 10 and 11; complexes 11, 14 and 15 were obtained with TtAgo containing substitutions of the catalytic residues in the active centre (acMut) (see Table S1 for details).

The key shown next to each structure indicates the lengths of the guide/target strands used for crystallization; positions of the guide strand paired with the target; location of the guide 3′-end (bound to PAZ or free); conformation of the glutamate finger in the active site (UP, ‘unplugged’; PI, ‘plugged in’); and the presence of catalytic Me^{2+} ions in the active centre.

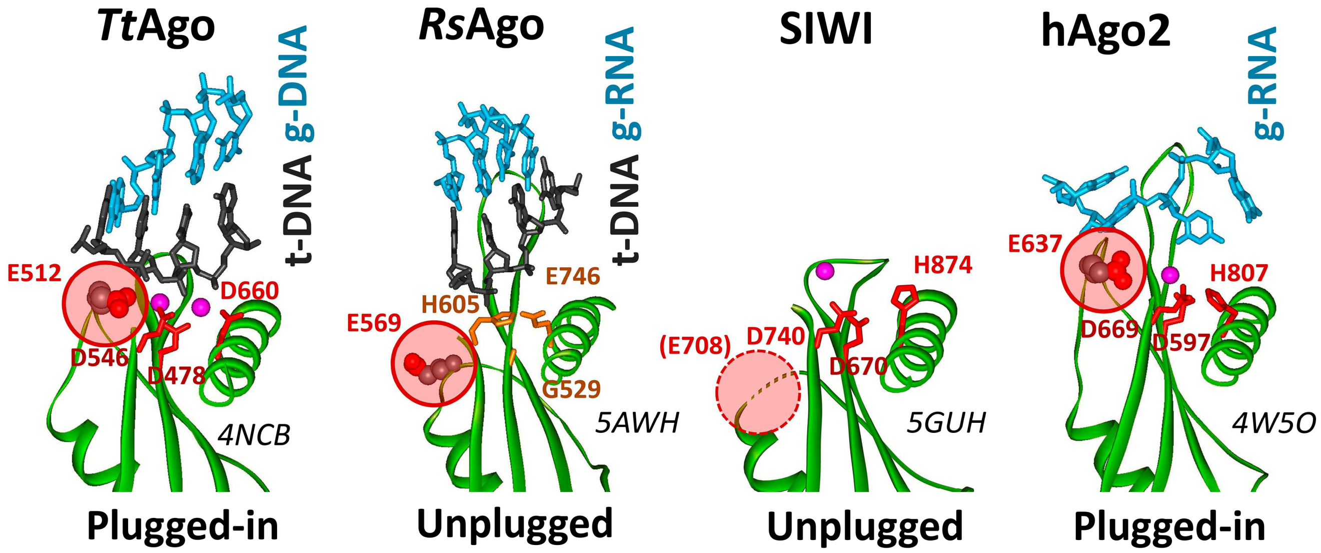

Supplementary Fig. 3. Variations in the active site conformations of pAgos and eAgos.
The glutamate finger is indicated with a red circle. The active site of TtAgo adopts the plugged-in conformation after target binding[2]. RsAgo contains substitutions of the catalytic residues (orange) and remains in the unplugged state even after target binding [6]. The SIWI protein is seen in the unplugged conformation in the absence of target RNA [7]. hAgo2 adopts the plugged-in state even in the absence of a target [8]. The PDB accession number is shown next to each structure

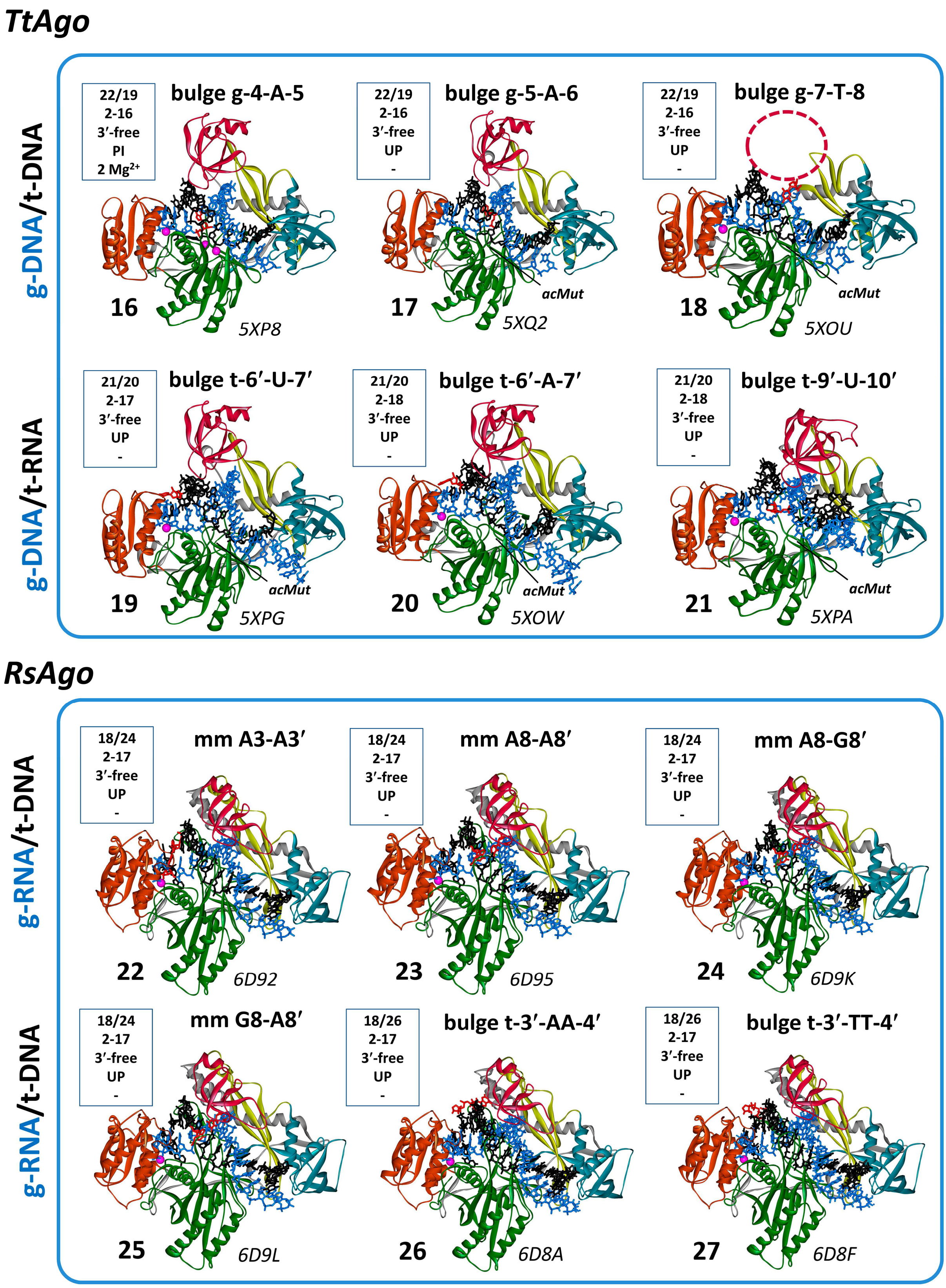

Supplementary Fig. 4. Structures of TtAgo[18] *(top)* and RsAgo[5] *(bottom)* ternary complexes with bulges and mismatches in the guide or target nucleic acid strands.

All designations correspond to Fig. 2. Positions and the identity of the bulged and mismatched nucleotides in each complex are indicated (*e.g.* g-4-A-5 corresponds to bulged A between 4th and 5th nucleotides of the guide strand; t-6′-U-7′ corresponds to bulged U between 6th and 7th nucleotides in the target strand; mm A3-A3’ corresponds to mismatch at position 3; *etc.*). Most complexes of TtAgo were obtained with the inactive D546N mutant (acMut). The PAZ domain was not resolved on the 5XOU structure.

Supplementary Table 1. Characteristics of published structures of pAgo and eAgo proteins and their complexes with nucleic acids

All listed structures are presented in Supplementary Fig. 1 (various pAgos and eAgos), Fig. 2 (compexes of TtAgo at different steps of its functional cycle) and Fig. 4 (ternary complexes of TtAgo and RsAgo with bulges and mismatches). The PDB accession number is shown for each complex; the numbers in bold correspond to complexes of TtAgo and RsAgo shown in Supplementary Fig. 2 and 4.

Ago/Host	Nucleic acid specificity *in vitro*		Structural features						Ref.
			Active site		MID-pocket	Bound nucleic acids		PDB ID	
	Guide	Target	Catalytic tetrad	Glu-finger position; Me^{2+}		g-DNA/ t-RNA	Scheme (guide strand, blue; target strand, black; unpaired bases, underlined; unmodeled nucleotides, grey)		
**AaAgo**<br><br>*Aquifex aeolicus*<br><br>hyper-thermophilic bacteria	DNA	RNA (DNA not tested)	$D^{502}E^{541}$ $D^{571}D^{683}$	Unplugged	Empty	None	–	1YVU	9
				Unplugged	Empty	Symmetric 22 nt RNA duplex	5′P-AGACAGCAUAUAUGCUGUCUUU-3′<br>3′-UUUCUGUCGUAUAUACGACAGA-P5′<br><br>Externally bound to the PAZ domain	2F8S	10
				Unplugged	Empty	Symmetric 26 nt RNA duplex	5′P-AGACAGCAUGCAUGCAUGCUGUCUUU-3′<br>3′-UUUCUGUCGUACGUACGUACGACAGA-P5′<br><br>Externally bound to the PAZ domain	2F8T	
				Unplugged	Empty	None	–	2NUB	11
**AfAgo**<br><br>*Archaeoglobus fulgidus*<br><br>hyper-thermophilic archaea	DNA (RNA)	DNA (RNA, lower affinity)	Inactive<br><br>(G^{186}, E^{221}, R^{257}, K^{394})	-	Empty	None	–	1W9H	12
				-	5′-P, Mn^{2+}	Symmetric 16 nt RNA duplex	5′P-UUCGACGCGUCGAAUU-3′<br>3′-UUAAGCUGCGCAGCUU-P5′	2BGG	13
				-	5′-P, Mg^{2+}	Symmetric 21 nt RNA duplex	5′P-AGACAGCAUUAUGCUGUCUUU-3′<br>3′-UUUCUGUCGUAUUACGACAGA-P5′	1YTU	14
				-	5′-P, Mn^{2+}	Symmetric 16 ntDNA duplex	5′P-TTCGACGCGTCGAATT-3′<br>3′-TTAAGCTGCGCAGCTT-P5′	2W42	15

**MpAgo**<br><br>*Marinitoga piezophila*<br><br>thermo-piezophilic bacteria	RNA	DNA (RNA, lower activity)	D^{446}, E^{482}, D^{515}, N^{624}	Unplugged	5′-OH	21 nt g-RNA	5′OH-UAUACAACCUACUACCUCAUU-3′	5I4A	16
				Unplugged	5′-OH	21 nt g-RNA/<br>21 nt t-DNA	5′OH-GGUACAACCUACUACCUCAUU-3′<br>3′-CCATGTTGGATGATGGAGTAA-P5′	5UX0	17
**MjAgo**<br><br>*Methanocaldococcus jannaschii*<br><br>hyper-thermophilic archaea	DNA	DNA	D^{504},E^{541}, D^{570}, D^{688}	Unplugged	Empty	None	-	5G5S	18
				Unplugged	5′-P, Mg^{2+}	21 nt g-DNA	5′P-TGAGGTAGTAGGTTGTATAGT-3′	5G5T	
**PfAgo**<br><br>*Pyrococcus furiosus*<br><br>hyper-thermophilic archaea	DNA	DNA	D^{558},E^{596}, D^{628},H^{745}	Unplugged	Empty	None	-	1U04	19
				Unplugged, Mn^{2+}	Empty	None	-	1Z25	20
				Unplugged	Empty	None	-	1Z26	
**TtAgo**<br><br>*Thermus thermophilus*<br><br>thermophilic bacteria	DNA	DNA (RNA, lower activity)	D^{478},E^{512}, D^{546},D^{660}	Unplugged	Empty	10 nt g-DNA	5′P-TGAGGTAGTA-3′	3DLB<br>**1**	1
				Unplugged	5′-P, Mg^{2+}	21 nt g-DNA	5′P-TGAGGTAGTAGGTTGTATAGT-3′	3DLH<br>**2**	
				Unplugged	5′-P, Mg^{2+}	21 nt g-DNA/<br>12 nt t-DNA	5′P-TGAGGTAGTAGGTTGTATAGT-3′<br>3′-GCTCCATCATCC-5′	4N47<br>**3**	2
				Unplugged	5′-P, Mg^{2+}	21 nt g-DNA/<br>15 nt t-DNA	5′P-TGAGGTAGTAGGTTGTATAGT-3′<br>3′-GCTCCATCATCCAAC-5′	4N41	

								**4**	
				Plugged in, 2 Mg^{2+}	5′-P, Mg^{2+}	21 nt g-DNA/ 19 nt t-DNA	5′P-TGAGGTAGTAGGTTGTATAGT-3′<br>3′-GCTCCATCATCCAACATAT-5′<br>^<br>Cleaved between positions t10’-t11’	4NCB **5**	
				Plugged in, 2 Mg^{2+}	5′-P, Mg^{2+}	21 nt g-DNA/ 16 nt t-DNA	5′P-TGAGGTAGTAGGTTGTATAGT-3′<br>3′-GCTCCATCATCCAACA-5′<br>^<br>Cleaved between positions t10’-t11’	4NCA **6**	
				Plugged in, 2 Mn^{2+}	5′-P, Mn^{2+}	21 nt g-DNA/ 19 nt t-DNA	5′P-TGAGGTAGTAGGTTGTATAGT-3′<br>3′-GCTCCATCATCCAACATAT-5′<br>^<br>Cleaved between positions t10’-t11’	4KPY **7**	
				Unplugged	5′-P, Mn^{2+}	21 nt g-DNA/ 19 nt t-DNA	5′P-TGAGGTAGTAGGTTGTATAGT-3′<br>3′-GCTCCATCAT<br>^<br>Cleaved between positions t10’-t11’ and preheated at 55°C before crystallization	4N76 **8**	
				Unplugged	5′-P, Mg^{2+}	21 nt g-DNA/ 20 nt t-RNA	5′P-TGAGGTAGTAGGTTGTATAGT-3′<br>3′-UGCUCCAUCACUCAACAUAU-5′<br><br>Mismatches at positions g10-g11	3HXM **9**	4
				Unplugged, Mg^{2+}	5′-P, Mg^{2+}	21 nt g-DNA/ 20 nt t-RNA	5′P-TGAGGTAGTAGGTTGTATAGT-3′<br>3′-UGCUCCAUCACUCAACAUAU-5′<br><br>Mismatches at positions g10-g11	3F73 **10**	3
				Unplugged, D546N mutant	5′-P, Mg^{2+}	21 nt g-DNA/ 12 nt t-RNA	5′P-TGAGGTAGTAGGTTGTATAGT-3′<br>3′-GCUCCAUCAUCC-5′	3HO1 **11**	4
				Plugged in, 2 Mg^{2+}	5′-P, Mg^{2+}	21 nt g-DNA/ 19 nt t-RNA	5′P-TGAGGTAGTAGGTTGTATAGT-3′<br>3′-GCUCCAUCAUCCAACAUAU-5′	3HVR **12**	

				Plugged in, Mg^{2+}	5′-P, Mg^{2+}	21 nt g-DNA/ 19 nt t-RNA	5′P-TGAGGTAGTAGGTTGTATAGT-3′<br>3′-GCUCCAUCAUCCAACAUAU-5′	3HM9<br>**13**	
				Plugged in, D546E mutant	5′-P, Mg^{2+}	21 nt g-DNA/ 15 nt t-RNA	5′P-TGAGGTAGTAGGTTGTATAGT-3′<br>3′-GCUCCAUCAUCCAAC-5′	3HJF<br>**14**	
				Plugged in, Mg^{2+}, D478N	5′-P, Mg^{2+}	21 nt g-DNA/ 19 nt t-RNA	5′P-TGAGGTAGTAGGTTGTATAGT-3′<br>3′-GCUCCAUCAUCCAACAUAU-5′	3HK2<br>**15**	
				Plugged in, 2 Mg^{2+}	5′-P, Mg^{2+}	22 nt g-DNA/ 19 nt t-DNA	**g-4-A-5**<br>5′P-TGAGAGTAGTAGGTTGTATAGT-3′<br>3′-GCTC_CATCATCCAACAUAU-5′<br><br>Weakened Watson-Crick pairs G6-C5	5XP8<br>**16**	21
				Unplugged, D546N mutant	5′-P	22 nt g-DNA/ 19 nt t-DNA	**g-5-A-6**<br>5′P-TGAGGATAGTAGGTTGTATAGT-3′<br>3′-GCTCC_ATCATCCAACAUAU-5′<br><br>Weakened Watson-Crick pairs G5-C5	5XQ2<br>**17**	
				Unplugged, D546N mutant	5′-P, Mg^{2+}	22 nt g-DNA/ 19 nt t-DNA	**g-7-T-8**<br>5′P-TGAGGTATGTAGGTTGTATAGT-3′<br>3′-GCTCCAT_CATCCAACAUAU-5′<br><br>Weakened Watson-Crick pairs A7-T7,G9-C8	5XOU<br>**18**	
				Unplugged, D546N mutant	5′-P, Mg^{2+}	21 nt g-DNA/ 20 nt t-RNA	**t-6′-U-7′**<br>5′P-TGAGGT_AGTAGGTTGTATAGT-3′<br>3′-GCUCCA ACAUCCAACAUAU-5′<br>U<br>Uracil loops-out of the duplex; non-canonical pairing A-A, weakened Watson-Crick pairs T6-A6	5XPG<br>**19**	
				Unplugged, D546N mutant	5′-P, Mg^{2+}	21 nt g-DNA/ 20 nt t-RNA	**t-6′-A-7′**<br>5′P-TGAGGT_AGTAGGTTGTATAGT-3′<br>3′-GCUCCA UCAUCCAACAUAU-5′<br>A<br>Adenine loops-out of the duplex, weakened Watson-Crick pairs T6-A6	5XOW<br>**20**	

				Unplugged, D546N mutant	5′-P, Mg^{2+}	21 nt g-DNA/ 20 nt t-RNA	**t-9′-U-10′**<br>5′P-TGAGGTAGT AGGTTGTATAGT-3′<br>3′-GCUCCAUCU GCCAACAUAU-5′<br>U<br>Uracil loops-out of the duplex	5XPA **21**	
**RsAgo**<br><br>*Rhodobacter sphaeroides*<br><br>proteobacteria	RNA	DNA	Inactive (G^{529}, E^{569}, H^{605}, E^{746})	Unplugged	5′-P, Mg^{2+}	18 nt g-RNA/ 18 nt t-DNA	5′P-UUACAACCUACUACCUCG-3′<br>3′-AATGTTGGATGATGGAGC-5′	5AWH	6
						18 nt g-RNA/ 24 nt t-DNA	5′P-UUACUGCACAGGUGACGA-3′<br>3′-GTCAATGACGTGTCCACTGCTGTC-5′	6D8P	5
						18 nt g-RNA/ 24 nt t-DNA	5′P-UUACUGCACAGGUGACGA-3′<br>3′-GTCAAGACGTGTCCACTGCTGTC-5′<br>Mismatches (A3·A3′ pair)	6D92 **22**	
						18 nt g-RNA/ 24 nt t-DNA	5′P-UUACUGCACAGGUGACGA-3′<br>3′-GTCAATGACGAGTCCACTGCTGTC-5′<br>Mismatches (A8·A8′ pair)	6D95 **23**	
						18 nt g-RNA/ 24 nt t-DNA	5′P-UUACUGCACAGGUGACGA-3′<br>3′-GTCAATGACGGGTCCACTGCTGTC-5′<br>Mismatches (A8·G8′ pair)	6D9K **24**	
						18 nt g-RNA/ 24 nt t-DNA	5′P-UUACUGCGCAGGUGACGA-3′<br>3′-GTCAATGACGAGTCCACTGCTGTC-5′<br>Mismatches (G8·A8′ pair)	6D9L **25**	
						18 nt g-RNA/ 26 nt t-DNA	**t-3′A4′, A5′**<br>5′P-UUA CUGCACAGGUGACGA-3′<br>3′-GTCAAT GACGTGTCCACTGCTGTC-5′<br>AA<br>Adenine loops-out of the duplex (A-A bulge)	6D8A **26**	
						18 nt g-RNA/ 26 nt t-DNA	**t-3′T4′, T5′**<br>5′P-UUA CUGCACAGGUGACGA-3′<br>3′-GTCAAT GACGTGTCCACTGCTGTC-5′<br>TT<br>Thymine loops-out of the duplex (T-T bulge)	6D8F **27**	

**hAgo1**<br>*Homo sapiens*	RNA	RNA	Inactive (D^{595}, E^{635}, D^{667}, R^{805})	Plugged in	5′-P Lys564	22 nt g-RNA	5′P-UGAGGUAGUAGGUUGUAUAGUU-3′	4KRF	22
**hAgo2**<br>*Homo sapiens*	RNA	RNA	D^{597},E^{637}, D^{669},H^{807}	Plugged in	5′-P, Lys566	20 nt miR-20a	5′P-UAAAGUGCUUAUAGUGCAGG	4F3T	23
				Plugged in; Mg^{2+}	5′-P, Lys566	21 nt g-RNA/ 11 nt t- RNA	5′P-UUCACAUUGCCCAAGUCUCUU-3′<br>3′-AAAAGUGUAAC-5′	4W5O	8
**hAgo3**<br>*Homo sapiens*	RNA	RNA	D^{598},E^{638}, D^{670},H^{808}	Plugged in	5′-P, Lys567	21 nt g-RNA	5′P-AAAAAAAA ... AAAAAUU-3′	5VM9	24
**KpAGO**<br>*Kluyveromyces polysporus*	RNA	RNA	D^{974},E^{1013}, D^{1046},D^{1198}	Plugged in	5′-P Lys939	8 nt guide RNA	5′P-UAAAAAAA-3′	4F1N	25
**SIWI**<br>*Bombyx mori*	RNA	RNA	D^{670},E^{708}, D^{740},H^{874}	Unplugged; Mg^{2+}	5′-P, Mg^{2+}	Endogenous 28 nt piRNA	5′P-UAUUU-5 ... 26-UUU$_m$-3′<br>Um - 2′-O-methylated U	5GUH	7

Supplementary References

1. Wang, Y., Sheng, G., Juranek, S., Tuschl, T. & Patel, D.J. Structure of the guide-strand-containing argonaute silencing complex. *Nature* **456**, 209-13 (2008).
2. Sheng, G. et al. Structure-based cleavage mechanism of *Thermus thermophilus* Argonaute DNA guide strand-mediated DNA target cleavage. *Proc Natl Acad Sci U S A* **111**, 652-7 (2014).
3. Wang, Y. et al. Structure of an argonaute silencing complex with a seed-containing guide DNA and target RNA duplex. *Nature* **456**, 921-6 (2008).
4. Wang, Y. et al. Nucleation, propagation and cleavage of target RNAs in Ago silencing complexes. *Nature* **461**, 754-61 (2009).
5. Liu, Y. et al. Accommodation of helical imperfections in *Rhodobacter sphaeroides* Argonaute ternary complexes with guide RNA and target DNA. *Cell Reports* **24**, 453-462 (2018).
6. Miyoshi, T., Ito, K., Murakami, R. & Uchiumi, T. Structural basis for the recognition of guide RNA and target DNA heteroduplex by Argonaute. *Nat Commun* **7**, 11846 (2016).

7. Matsumoto, N. et al. Crystal Structure of Silkworm PIWI-Clade Argonaute Siwi Bound to piRNA. *Cell* **167**, 484-497 e9 (2016).
8. Schirle, N.T., Sheu-Gruttadauria, J. & MacRae, I.J. Structural basis for microRNA targeting. *Science* **346**, 608-13 (2014).
9. Yuan, Y.R. et al. Crystal structure of A. aeolicus argonaute, a site-specific DNA-guided endoribonuclease, provides insights into RISC-mediated mRNA cleavage. *Mol Cell* **19**, 405-19 (2005).
10. Yuan, Y.R., Pei, Y., Chen, H.Y., Tuschl, T. & Patel, D.J. A potential protein-RNA recognition event along the RISC-loading pathway from the structure of *A. aeolicus* Argonaute with externally bound siRNA. *Structure* **14**, 1557-65 (2006).
11. Rashid, U.J. et al. Structure of *Aquifex aeolicus* argonaute highlights conformational flexibility of the PAZ domain as a potential regulator of RNA-induced silencing complex function. *J Biol Chem* **282**, 13824-32 (2007).
12. Parker, J.S., Roe, S.M. & Barford, D. Crystal structure of a PIWI protein suggests mechanisms for siRNA recognition and slicer activity. *EMBO J* **23**, 4727-37 (2004).
13. Parker, J.S., Roe, S.M. & Barford, D. Structural insights into mRNA recognition from a PIWI domain-siRNA guide complex. *Nature* **434**, 663-6 (2005).
14. Ma, J.B. et al. Structural basis for 5'-end-specific recognition of guide RNA by the *A. fulgidus* Piwi protein. *Nature* **434**, 666-70 (2005).
15. Parker, J.S., Parizotto, E.A., Wang, M., Roe, S.M. & Barford, D. Enhancement of the seed-target recognition step in RNA silencing by a PIWI/MID domain protein. *Mol Cell* **33**, 204-14 (2009).
16. Kaya, E. et al. A bacterial Argonaute with noncanonical guide RNA specificity. *Proc Natl Acad Sci U S A* **113**, 4057-62 (2016).
17. Doxzen, K.W. & Doudna, J.A. DNA recognition by an RNA-guided bacterial Argonaute. *PLoS One* **12**, e0177097 (2017).
18. Willkomm, S. et al. Structural and mechanistic insights into an archaeal DNA-guided Argonaute protein. *Nat Microbiol* **2**, 17035 (2017).
19. Song, J.J., Smith, S.K., Hannon, G.J. & Joshua-Tor, L. Crystal structure of Argonaute and its implications for RISC slicer activity. *Science* **305**, 1434-7 (2004).
20. Rivas, F.V. et al. Purified Argonaute2 and an siRNA form recombinant human RISC. *Nat Struct Mol Biol* **12**, 340-9 (2005).
21. Sheng, G. et al. Structure/cleavage-based insights into helical perturbations at bulge sites within *T. thermophilus* Argonaute silencing complexes. *Nucleic Acids Res* **45**, 9149-9163 (2017).
22. Faehnle, C.R., Elkayam, E., Haase, A.D., Hannon, G.J. & Joshua-Tor, L. The making of a slicer: activation of human Argonaute-1. *Cell Rep* **3**, 1901-9 (2013).
23. Elkayam, E. et al. The structure of human argonaute-2 in complex with miR-20a. *Cell* **150**, 100-10 (2012).
24. Park, M.S. et al. Human Argonaute3 has slicer activity. *Nucleic Acids Res* **45**, 11867-11877 (2017).
25. Nakanishi, K., Weinberg, D.E., Bartel, D.P. & Patel, D.J. Structure of yeast Argonaute with guide RNA. *Nature* **486**, 368-74 (2012).
